# Quantitative ultrasound measurements of bone strength in female adolescent idiopathic scoliosis patients

**DOI:** 10.1186/1748-7161-8-S1-O7

**Published:** 2013-06-03

**Authors:** H Wang, Q Du, PJ Chen, JA Li, XH He

**Affiliations:** 1 Xinhua Hospital, Shanghai, China; 2 Shanghai University of Sports, Shanghai, China; 3 Jiangsu Province People's hospital, Jiangshu, China; 4 Palmer College of Chiropractic, Florida, USA

## Background

The etiology of adolescent idiopathic scoliosis (AIS) has been linked to many factors, such as asymmetric growth, neuromuscular conditions and genetics. Several studies have been focused on the relationship between bone and scoliosis [1,2]. However, very few studies have examined the effect of AIS on bone mineralization.

## Aim

The purpose of this study was to determine bone strength by quantitative ultrasound (QUS) measurements of bone speed of sound (SOS) along the longitudinal axis of the radial bones in scoliosis children.

## Methods

This study was approved by the Human Research Ethic Committee of the School of Medicine, Jiaotong University, China. Eighty nine untreated female AIS patients were recruited in this study. The age range was from 10 to 16 years. Those who were using calcium supplements and those who had history of bone diseases were excluded from the study. The diagnoses of all AIS were confirmed by radiography based on American College of Radiology Guidelines and Recommendations. Cobb’s angles ranged from 10-52° (average 27.13°). The types of scoliosis included thoracic, lumbar, thoracolumbar and double curvatures. Patients’ menstrual conditions and family history were also taken in consideration. SOS was measured (by Sunlight omnisense) in the distal 1/3 of the radial bones. Data were compared to age and gender-matched norms (standard SOS value provided by Sunlight system).

## Results

Radial SOS was significantly reduced in AIS patients compared to non-AIS subjects (P < 0.01); the radial SOS was also correlated to the age of the onset of menstrual cycle (lower in pre-menstrual cycle patients, 3626.92±124.35 vs. 3702.68±192.23, P<0.05). No significant correlations were found among the type of curvatures, the degree of Cobb’s angle, family history and bone SOS (P < 0.05).

## Conclusion

Bone strength measured by QUS is reduced in AIS patients. The onset of menstrual cycle may have an effect on the reduction of SOS. Bone SOS is not affected by the types and severity of the scoliosis.
